# A randomized crossover pilot study comparing the efficacy of an auto-demand oxygen delivery system with that of a conventional demand oxygen delivery system in patients with chronic respiratory failure

**DOI:** 10.1097/MD.0000000000027191

**Published:** 2021-09-17

**Authors:** Takehiro Otoshi, Tatsuya Nagano, Sae Murakami, Takashi Omori, Daisuke Hazama, Naoko Katsurada, Masatsugu Yamamoto, Motoko Tachihara, Yoshihiro Nishimura, Kazuyuki Kobayashi

**Affiliations:** a Division of Respiratory Medicine, Department of Internal Medicine, Kobe University Graduate School of Medicine, 7-5-1 Kusunokicho, Chuo-ku, Kobe, Hyogo, Japan; b Clinical and Translational Research Center, Kobe University Hospital, 7-5-2 Kusunokicho, Chuo-ku, Kobe, Hyogo, Japan.

**Keywords:** chronic respiratory failure, demand oxygen delivery system, portable oxygen concentrator, trigger sensitivity

## Abstract

**Introduction:**

: When using portable oxygen, a demand oxygen delivery system (DODS), which senses the beginning of inhalation and delivers a bolus of oxygen, is often used. However, conventional DODS may not supply sufficient oxygen when reduced tidal flow fails to trigger the flow sensor. Recently, “auto-DODS,” which detects the negative pressure of inhalation and switches among 3 trigger sensitivity levels (*standard*, *high,* and *extra high*), has been developed to improve the efficacy of oxygenation. An auto-DODS can also supply pulsed-flow oxygen when it detects apnea, whereas a conventional DODS has only *standard* sensitivity. This randomized, open-label, crossover pilot study compared the performance of an auto-DODS with that of a conventional DODS.

**Methods:**

: We recruited patients with chronic obstructive pulmonary disease (COPD) or interstitial pneumonia receiving long-term oxygen therapy. Interventions were performed on 2 different days for each participant. On each day, an auto-DODS or a conventional DODS were tested at rest for 30 minutes and during the 6-minute walk test. The primary outcome was mean oxygen saturation (SpO2). Secondary outcomes were the ratios of time for each sensitivity level and pulsed-flow oxygen when using the auto-DODS, total time desaturated below SpO2 90%, percentage of time desaturated below SpO2 90%, minimum SpO2, mean and maximum pulse rate, six-minute walk distance, recovery time after 6-minute walk test, modified Borg scale, comfort, and discomfort index.

**Results:**

: When using the auto-DODS at rest, a *high* or *extra high* sensitivity level was observed in addition to *standard* sensitivity in 6 of 8 participants. During the 6-minute walk test, only *standard* sensitivity was observed in 6 participants. Mean SpO2 differences between the auto-DODS and conventional DODS at rest and during the 6-minute walk test were −0.6 [−4.5, 3.4] and 0.0 [−2.5, 2.5] ([95% confidence interval]), respectively, neither of which were significant (*P* = .73 and *P* = .99). There were no significant differences in secondary outcomes. There were no adverse events when using the auto-DODS.

**Conclusions:**

: This study showed that the auto-DODS did not show superiority in oxygenation either at rest or during exercise compared to a conventional DODS. The auto-DODS was shown to supply oxygen safely and detect inhalations with various trigger sensitivities.

## 1 Introduction

Long-term oxygen therapy is currently provided for many patients with chronic respiratory failure, such as chronic obstructive pulmonary disease (COPD). It has already been proven that long-term oxygen therapy can reverse the progression of pulmonary hypertension and improve the quality of life and prognosis of patients with COPD.^[1–3]^

Portable oxygen is also prescribed to an increasing number of hypoxemic patients.^[3]^ It is still not clear whether the use of portable oxygen itself improves the prognosis of hypoxemic patients, but it has already been shown that portable oxygen helps to relieve the symptoms of breathlessness during exertion, improve mobility and increase compliance with oxygen therapy.^[4–6]^

When using portable oxygen, a demand oxygen delivery system (DODS) that senses the beginning of inhalation and immediately delivers a short bolus of oxygen (limiting oxygen flow to the onset of inhalation) is often used because this system limits oxygen consumption and extends the time that patients can use an oxygen cylinder in an ambulatory setting.^[3]^ However, there is a concern that a DODS may not supply sufficient oxygen because reduced tidal flow may fail to trigger the flow sensor.^[4]^

To solve this problem, a new type of portable oxygen concentrator with an “auto-DODS,” which can detect the negative pressure of inhalation and switch among 3 trigger sensitivities (*standard*, *high,* and *extra high*), has been developed recently to improve the efficacy of oxygenation in hypoxemic patients. This oxygen concentrator can also supply pulsed-flow oxygen when it detects apnea. Thus, we hypothesized that this auto-DODS is superior to a conventional DODS in improving oxygenation, and the present study aims to compare the performance of an auto-DODS with that of a conventional DODS both at rest and during exertion.

## 2 Methods

### 2.1 Design

This clinical trial is a randomized, open-label, crossover, pilot study intending to compare the efficacy of an auto-DODS with that of a conventional DODS in patients with chronic respiratory failure at rest and during the 6-minute walk test. This trial was performed at Kobe University Hospital and was approved by the Kobe University Clinical Research Ethical Committee (permission number: C190013). This trial was also registered in the Japan Registry of Clinical Trials (jRCT) on August 23, 2019 (trial ID: jRCTs052190041). The registration period was set from August 2019 to April 2020. The follow-up period was 2 months from the date when the final case was registered. The study protocol was reported by Nagano et al.^[7]^

Written informed consent was obtained from all the participants included in this study. All procedures performed were in accordance with the ethical standards of the institutional and national committees and with the 1964 Helsinki Declaration and its later amendments or comparable ethical standards.

### 2.2 Participants

We recruited patients aged 20 years or older with COPD or interstitial pneumonia who received long-term oxygen therapy (less than 4 L/min during the daytime). Detailed inclusion and exclusion criteria are summarized in a previous paper of this study protocol,^[7]^ or mentioned in the jRCT (trial ID: jRCTs052190041).

### 2.3 Randomization and masking

Interventions were performed on 2 different days for each participant: the first day of testing (Day 1), at a 4-week interval, and on another day of testing (Day 29). On Day 1 and Day 29, the auto-DODS or conventional DODS were tested both at rest for 30 minutes and during the 6-minute walk test. The oxygen flow rate during these tests was adjusted based on the prescription flow rate in participants’ daily lives, and the flow rate was set to be the same for both the auto-DODS and conventional DODS.

After registration, participants were randomly assigned to either Group 1 (an auto-DODS on Day 1 followed by a conventional DODS on Day 29) or Group 2 (a conventional DODS on Day 1 followed by an auto-DODS on Day 29) (allocation ratio 1:1). A detailed study protocol that describes randomization and masking procedures has been mentioned by Nagano et al.^[7]^

### 2.4 Sample size

Because no prior study has been conducted in hypoxemic patients using an auto-DODS, it was not possible to statistically ascertain the required number of participants in this trial. However, the sample size was determined to be 26 cases (13 cases per group) in consideration of feasibility within the study period. Because this is a pilot study, we decided that the registration period won’t be extended even if the sample size was not fulfilled during the period.^[7]^

### 2.5 Device and measurement

On Day 1 and Day 29, a Hi-Sanso Portable αII oxygen concentrator equipped with auto-DODS and conventional DODS settings (Teijin Pharma Ltd., Tokyo, Japan) was used. Through a nasal cannula, the auto-DODS senses the negative pressure of inhalation and switches its trigger sensitivity among 3 levels (*standard*, *high*, and *extra high*: the higher sensitivity level can detect shallower inhalation), and thereupon directs the solenoid valve to open momentarily to deliver a bolus of oxygen. Specifically, the negative pressure of inhalation detected by high sensitivity is half that of the pressure detected by standard sensitivity, while the pressure detected by extra high sensitivity is one tenth of the pressure detected by standard sensitivity. With higher sensitivity level, oxygen can be supplied even during shallower inhalation. The trigger sensitivity is adjusted automatically; when the valve opens fewer than a predetermined number of times during a fixed interval, the sensitivity increases by 1 step, and when the valve opens more than the predetermined number of times, the sensitivity decreases by 1 step. Additionally, the auto-DODS supplies pulsed-flow oxygen when it detects apnea. The conventional DODS senses the negative pressure of inhalation with *standard* sensitivity only, and it does not respond to apnea (it only sounds the apnea alarm).

During measurement at rest, each participant, equipped with either of the DODS, was asked to sit on a chair, and both oxygen saturation (SpO2) and pulse rate were continuously recorded for 30 minutes by a pulse oximeter, PULSOX-Me300 (KONICA MINOLTA, Inc., Tokyo, Japan). During the 6-minute standardized walk test, participants were also equipped with either of the DODS, and both SpO2 and pulse rate were similarly recorded. Apnea alarms were also recorded during the measurement time.

### 2.6 Outcomes

The primary outcomes were mean SpO2 at rest and during the 6-minute walk test. The secondary outcomes were

1. the ratios of time for each sensitivity and pulsed-flow oxygen when using the auto-DODS (percentage of equipment time spent on each sensitivity level),
2. total time desaturated below SpO2 90% at rest and during the 6-minute walk test,
3. percentage of time desaturated below SpO2 90% at rest and during the 6-minute walk test,
4. minimum value of SpO2 at rest and during the 6-minute walk test,
5. mean pulse rate at rest and during the 6-minute walk test,
6. maximum pulse rate at rest and during the 6-minute walk test,
7. six-minute walk distance,
8. recovery time (the time needed to recover SpO2 after the 6-minute walk test),
9. modified Borg scale (the difference between the worst score during the 6-minute walk test and the score before the test),
10. comfort when using the DODS measured by a numerical rating scale (from 0–10, 0 is very uncomfortable and 10 is very comfortable) and
11. discomfort index when using DODS measured by questionnaires (from 5–20, 5 is very comfortable and 20 is very uncomfortable).

The details of the discomfort index questionnaires are shown in the Supplemental Digital Content.

### 2.7 Data presentation and statistical analysis

Data are summarized using the median (range) or mean (standard deviation) for continuous variables and numbers for categorical variables. The differences in outcomes between the auto-DODS and conventional DODS and their 95% confidence intervals (CIs) were also calculated. *P* values were calculated based on a *t*-test. Additional details were described by Nagano et al.^[7]^ In all cases, *P* values ≤.05 were considered significant. Analyzes were carried out using SAS Proprietary Software 9.4, SAS/STAT 15.1, and JMP 9.0.2 (SAS Institute Inc., Cary, NC). Statistical analyses were performed by a biostatistician at the Clinical and Translational Research Center at Kobe University Hospital (S.M.).

## 3 Results

### 3.1 The result of randomization

**Figure 1:**
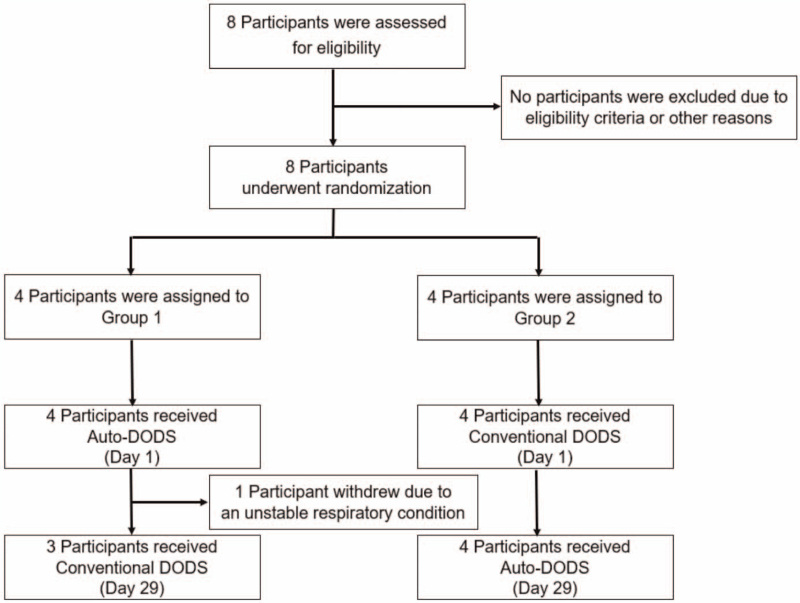
Participant randomization. In this trial, 8 participants were assessed for eligibility. None of them were excluded due to eligibility criteria or other reasons, and all of them underwent randomization. Four participants were assigned to Group 1, and 4 were assigned to Group 2. In Group 1, 4 received auto-DODS treatment on day 1, while 3 of them received conventional DODS treatment on day 29 (1 participant was withdrawn from the study because of an unstable respiratory condition according to the judgment of a principal investigator). In Group 2, 4 patients received conventional DODS treatment on day 1, and all of them received auto-DODS treatment on day 29. DODS = demand oxygen delivery system.

During the registration period, 8 participants were included in this trial. The flow chart of participant randomization is shown in Figure 1. In this trial, 4 participants were assigned to each group. In Group 1, 4 participants received auto-DODS treatment on day 1, while 3 of them received conventional DODS treatment on day 29 (1 participant was withdrawn from the study because of an unstable respiratory condition according to the judgment of a principal investigator). In Group 2, 4 participants received conventional DODS treatment on day 1, and all received auto-DODS treatment on day 29.

### 3.2 Participants’ characteristics

The characteristics of the participants in this study are summarized in Table 1. The ages of participants in Group 1 were higher than those of participants in Group 2, but between the 2 groups, there were no differences in the other variables (sex, body mass index, underlying disease, hemoglobin, FEV_1_% predicted, FVC % predicted, oxygen flow at rest, and oxygen flow during exertion).

**Table 1:** Participants’ characteristics.

	Group 1 (Auto-DODS followed by conventional DODS)	Group 2 (Conventional DODS followed by auto-DODS)	Total participants (Group 1 + Group 2)
Subjects, n	4	4	8
Age, yrs	80 (59–89)^∗^	69 (46–85)^∗^	74 (46–89)^∗^
Sex (male), n	3	3	6
BMI	21.9 (14.8–23.8)^∗^	22.3 (17.2–25.5)^∗^	22.2 (14.8–25.5)^∗^
Underlying disease (COPD/IP, n)	3/1	3/1	6/2
Hb	14.3 (10.0–15.3)^∗^	14.5 (13.4–16.1)^∗^	14.5 (10.0–16.1)^∗^
FEV_1_% predicted	68.8 (32.9–88.4)^∗^	66.6 (32.1–83.7)^∗^ (n = 3)^∗∗^	66.6 (32.1–88.4)^∗^ (n = 7)^∗∗^
FVC % predicted	81.2 (70.9–95.4)^∗^	92.5 (65.1–95.1)^∗^ (n = 3)^∗∗^	82.1 (65.1–95.4)^∗^ (n = 7)^∗∗^
Oxygen flow at rest (L/min)	1.5 (1.0–2.0)^∗^	2.5 (1.0–3.0)^∗^	2.0 (1.0–3.0)^∗^
Oxygen flow during exertion (L/min)	2.5 (1.0–4.0)^∗^	3.0 (2.0–3.0)^∗^	3.0 (1.0–4.0)^∗^

BMI = body mass index, COPD = chronic obstructive pulmonary disease, DODS = demand oxygen delivery system, FEV_1_ = forced expiratory volume in one second, FVC = forced vital capacity, Hb = haemoglobin, IP = interstitial pneumonia.

∗ Numbers indicate median (minimum-maximum).

∗∗ Numbers in parentheses indicate the numbers of patients with data available.

### 3.3 The ratios of time for each sensitivity level and pulsed-flow oxygen when using the auto- demand oxygen delivery system

**Figure 2:**
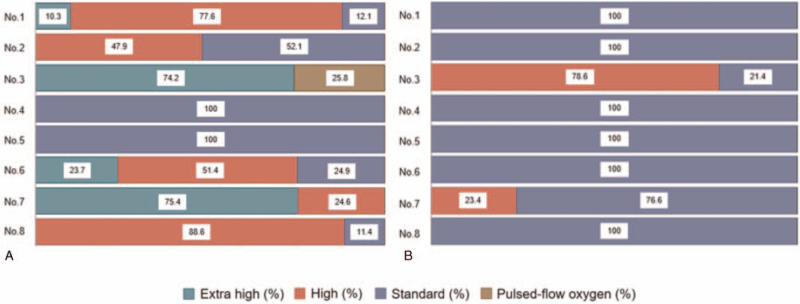
The ratios of time for each sensitivity level (*standard*, *high* and *extra high*) and pulsed-flow oxygen when using the auto-demand oxygen delivery system. (A) The ratios at rest among 8 participants (from No. 1 to No. 8). (B) The ratios during the 6-minute walk test among 8 participants. Green, red, blue, and brown bars indicate *extra high*, *high,* and *standard* sensitivity levels and pulsed-flow oxygen, respectively.

The ratios of time at each sensitivity level (*standard*, *high,* and *extra high*) and pulsed-flow oxygen when using the auto-DODS are shown in Figure 2A (at rest) and Figure 2B (during the 6-minute walk test). For participant No. 1 in Figure 2A, the sensitivity was recorded for only half of the measurement time (15 minutes) due to recording trouble. The auto-DODS can supply pulsed-flow oxygen when it detects apnea, and pulsed-flow oxygen was observed in participant No. 3 at rest (Fig. 2A).

At rest, in addition to *standard* sensitivity, *high* or *extra high* sensitivity was observed in 6 of the 8 participants. However, during the 6-minute walk test, only *standard* sensitivity was observed in 6 of the 8 participants.

### 3.4 Comparison between the auto- demand oxygen delivery system and conventional demand oxygen delivery system at rest

As mentioned above (in Fig. 1), in Group 1, 1 participant did not receive conventional DODS treatment. Therefore, the data of that participant (participant No. 3 in Fig. 2) were excluded from the following analyses. Table 2 contains data from the remaining 7 participants, and primary outcomes (mean SpO2) and secondary outcomes (total time desaturated below SpO2 90%, percentage of time desaturated below SpO2 90%, minimum SpO2, mean pulse rate per minute, maximum pulse rate per minute, comfort when using the DODS measured by numerical rating scale, discomfort index value when using the DODS measured by questionnaires) at rest are shown. The differences in outcomes between the auto-DODS and conventional DODS and their 95% CIs are also shown in Table 2. For mean SpO2, the difference between the auto-DODS and conventional DODS was −0.6 [−4.5, 3.4] (the estimate and [95% CI]), and it was not statistically significant (*P* = .73). There were also no significant differences between the auto-DODS and conventional DODS in any of the secondary outcomes.

**Table 2:** Comparison between the auto-demand oxygen delivery system and conventional demand oxygen delivery system at rest.

			Auto-DODS – Conventional DODS (Difference in outcomes) (n = 7)		
	Auto-DODS (n = 7)	Conventional DODS (n = 7)	Estimates	[95% CI]	*P* value
Mean SpO2, %	95.5 (2.7)^∗^	96.3 (2.3)^∗^	−0.6	[−4.5, 3.4]	.73
Total time desaturated below SpO2 90%, seconds	82 (217)^∗^	13 (34)^∗^	57	[−163, 277]	.54
Percentage of time desaturated below SpO2 90%, %	5 (12)^∗^	1 (2)^∗^	3	[−9, 15]	.54
Minimum SpO2, %	93.1 (2.9)^∗^	93.7 (2.4)^∗^	−0.5	[−4.8, 3.8]	.78
Mean pulse rate per minute	72 (12)^∗^	71 (11)^∗^	0	[−6, 6]	.99
Maximum pulse rate per minute	82 (15)^∗^	80 (12)^∗^	2	[−3, 7]	.41
Comfort when using the DODS measured by NRS	9 (1)^∗^	9 (1)^∗^	0	[−0.5, 0.6]	.85
Discomfort index when using the DODS measured by questionnaire	7 (2)^∗^	7 (3)^∗^	0	[−3, 3]	1.0

CI = confidence interval, DODS = demand oxygen delivery system, NRS = numerical rating scale (from 0 to 10, 0 is very uncomfortable and 10 is very comfortable).

∗ Number indicates mean (standard deviation).

Moreover, when using the conventional DODS at rest, apnea alarms were recorded in only 1 participant. This participant had a diagnosis of COPD. While his body mass index and FVC % predicted were 22.4 and 82.1% (both in the normal range), he had chronic heart failure as an underlying illness. In this participant, when using the auto-DODS, *extra high* and *high* sensitivity accounted for 75.4% and 24.6% of the measurement time, respectively (participant No. 7 in Fig. 2). Additionally, in this participant, the auto-DODS showed better results than the conventional DODS with regard to mean SpO2 and other secondary outcomes (Table 3).

**Table 3:** Comparison between the auto-demand oxygen delivery system and conventional demand oxygen delivery system at rest in a participant.

	Auto-DODS	Conventional DODS
Apnea alarms	Absent	Present
The ratio of time for each sensitivity level	*Extra high* 75.4%, *high* 24.6%	*Standard* 100%
Mean SpO2, %	94.5	92.1
Total time desaturated below SpO2 90%, second	0	89
Percentage of time desaturated below SpO2 90%, %	0	5
Minimum SpO2, %	93.2	89.1
Mean pulse rate per min	54	62
Maximum pulse rate per min	63	68
Comfort when using the DODS measured by NRS	10	9
Discomfort index when using the DODS measured by questionnaire	7	13

DODS = demand oxygen delivery system, NRS = numerical rating scale (from 0 to 10, 0 is very uncomfortable and 10 is very comfortable).

### 3.5 Comparison between the auto-demand oxygen delivery system and conventional demand oxygen delivery system during the 6-minutewalk test

Table 4 shows the primary outcomes (mean SpO2) and secondary outcomes during the 6-minute walk test. The differences in outcomes between the auto-DODS and conventional DODS and their 95% CIs are also shown in Table 4. For mean SpO2, the difference between the auto-DODS and conventional DODS was 0.0 [−2.5, 2.5] (the estimate and [95% CI]), and it was not statistically significant (*P* = .99). There were also no significant differences between the auto-DODS and conventional DODS in any of the secondary outcomes.

**Table 4:** Comparison between the auto-demand oxygen delivery system and conventional demand oxygen delivery system during the 6-minute walk test.

			Auto-DODS – Conventional DODS (Difference in outcomes) (n = 7)		
	Auto-DODS (n = 7)	Conventional DODS (n = 7)	Estimates	[95% CI]	*P* value
Mean SpO2, %	87.1 (8.3)^∗^	87.1 (8.5)^∗^	0.0	[−2.5, 2.5]	.99
Total time desaturated below SpO2 90%, second	180 (116)^∗^	185 (113)^∗^	−10	[−119, 98]	.82
Percentage of time desaturated below SpO2 90%, %	50 (32)^∗^	51 (31)^∗^	−3	[−33, 27]	.82
Minimum SpO2, %	80.1 (12.0)^∗^	80.9 (12.4)^∗^	−0.3	[−2.9, 2.4]	.82
Mean pulse rate per min	100 (11)^∗^	99 (11)^∗^	1	[−7, 9]	.79
Maximum pulse rate per min	114 (11)^∗^	110 (12)^∗^	3	[−6, 12]	.45
Six-min walk distance	264 (105)^∗^	241 (126)^∗^	21	[−25, 66]	.30
Recovery time, second	173 (88)^∗^	143 (98)^∗^	33	[−19, 84]	.17
Modified Borg scale^∗∗^	4 (2)^∗^	4 (2)^∗^	0	[−0.5, 1.3]	.29
Comfort when using the DODS measured by NRS	9 (2)^∗^	7 (3)^∗^	1	[−2, 5]	.41
Discomfort index when using the DODS measured by questionnaire	9 (3)^∗^	10 (5)^∗^	−1	[−7, 5]	.69

CI = confidence interval, DODS = demand oxygen delivery system, NRS numerical rating scale (from 0 to 10, 0 is very uncomfortable and 10 is very comfortable).

∗ Numbers indicate means (standard deviation).

∗∗ The difference between the worst score during the 6-minute walk test and the score before the test.

### 3.6 Safety when using the demand oxygen delivery system

There were no adverse events or problems when using the auto-DODS and conventional DODS in this trial.

## 4 Discussion

Home oxygen therapy is an established treatment for hypoxemic patients. In recent years, an increasing number of patients with chronic respiratory diseases, such as COPD, interstitial pneumonia, and lung cancer, have been receiving home oxygen therapy.^[8]^ Moreover, the Japanese respiratory society reported that among patients using home oxygen therapy, more than 70% of them are using the DODS in an ambulatory setting.

There is a concept of “fast space ventilation” and “slow space ventilation”.^[3]^ According to this concept, fast space fills during the earliest part of inhalation and is well perfused with pulmonary circulation. On the other hand, slow space is filled at the very end of inhalation and is poorly perfused.^[3]^ Based on this concept, it is thought that providing oxygen flow at the onset of inhalation by the DODS leads to more efficient arterial oxygenation. Moreover, a majority of the respiratory cycle is spent in exhalation, while most of the time during inhalation is spent filling the dead space.^[3]^ Therefore, the concept of the DODS is very reasonable.

In a previous study, it was reported that the DODS can maintain arterial oxygenation equivalent to continuous oxygen flow both at rest and during exercise in hypoxemic patients with COPD.^[9]^ However, in another study, Roberts et al showed that among subjects with severe COPD whose oxygen saturation fell below 90% during exercise, the DODS was less effective for oxygenation during walking than continuous oxygen flow.^[4]^ We thought that reduced tidal flow or rapid respiratory rates during rest or exercise could fail to trigger the flow sensor when using the DODS. Therefore, in this trial, we tested whether the auto-DODS, which can detect the negative pressure of inhalation and switch among 3 trigger sensitivity levels (*standard*, *high,* and *extra high*), could improve oxygenation compared to the conventional DODS, which senses the negative pressure of inhalation with *standard* sensitivity only.

At rest, in addition to *standard* sensitivity, *high* or *extra high* sensitivity was observed in 6 of the 8 participants when using the auto-DODS. However, in contrast to our hypothesis, there were no significant differences between the auto-DODS and conventional DODS in mean SpO2 or any of the other variables. The estimates indicated as differences between the 2 DODSs in mean SpO2, considering their 95% CI, were approximately zero, as shown in Table 2. From the results of this study, we can conclude that increasing the trigger frequency with the auto-DODS does not improve oxygenation at rest. Interestingly, however, as shown in Table 3, in 1 participant with shallow breathing at rest (apnea alarms were recorded only in this participant when using conventional DODS), higher trigger sensitivities with the auto-DODS prevented trigger failure and improved oxygenation compared to the conventional DODS. This result leads to the conclusion that the higher trigger sensitivity levels (*extra high* and *high*) with the auto-DODS could prevent trigger failure that would have happened with the conventional DODS. Thus, among patients with shallow breathing at rest, the auto-DODS may show superiority for improving oxygenation compared to the conventional DODS, and this should be elucidated in future studies.

During the 6-minute walk test, only *standard* sensitivity was observed in 6 out of 8 participants when using the auto-DODS. This means that flow sensors can be successfully triggered during exercise in most of the participants by using only *standard* sensitivity and that the auto-DODS is unlikely to have an advantage in triggering flow sensors over the conventional DODS during exercise. Additionally, during the 6-minute walk test, no significant difference was observed in SpO2 between the auto-DODS and conventional DODS.

The limitations of the current clinical trial are as follows. First, the number of participants was small (8 participants). Although it was not possible to statistically determine the required number of participants because of the exploratory design of this trial, further studies with more subjects are needed to confirm our findings. Second, oxygenation of the participants was assessed not by arterial oxygen saturation or arterial oxygen pressure but by SpO2 measured by a pulse oximeter. However, pulse oximetry has been proven to be effective in measuring arterial desaturation, and we think that this limitation has no serious effect on the results of this study.^[10]^

In conclusion, this clinical trial has shown that the utility of the auto-DODS in maintaining oxygenation at rest and during exercise was equal to that of the conventional DODS among patients with chronic respiratory failure. Additionally, the auto-DODS was demonstrated to be safe when used for hypoxemic patients both at rest and during exercise.

## Acknowledgments

The authors thank the staff of the Clinical and Translational Research Center of Kobe University Hospital for their support in this trial. The authors also thank the staff of the Division of Respiratory Medicine of Kobe University Hospital for recruiting and caring for patients in this trial. The authors also thank Springer Nature Author Services for editing our manuscript for proper English language.

## Author contributions

**Conceptualization:** Takehiro Otoshi, Tatsuya Nagano, Sae Murakami, Daisuke Hazama, Naoko Katsurada, Masatsugu Yamamoto, Motoko Tachihara, Yoshihiro Nishimura, Kazuyuki Kobayashi.

**Data curation:** Takehiro Otoshi, Tatsuya Nagano, Sae Murakami, Takashi Omori.

**Formal analysis:** Takehiro Otoshi, Tatsuya Nagano, Sae Murakami, Takashi Omori.

**Funding acquisition:** Tatsuya Nagano, Yoshihiro Nishimura.

**Investigation:** Takehiro Otoshi, Tatsuya Nagano, Sae Murakami, Takashi Omori, Daisuke Hazama, Naoko Katsurada, Masatsugu Yamamoto, Motoko Tachihara, Yoshihiro Nishimura, Kazuyuki Kobayashi.

**Methodology:** Takehiro Otoshi, Tatsuya Nagano, Sae Murakami, Takashi Omori, Yoshihiro Nishimura.

**Project administration:** Tatsuya Nagano, Yoshihiro Nishimura.

**Resources:** Tatsuya Nagano.

**Software:** Tatsuya Nagano, Sae Murakami, Takashi Omori.

**Supervision:** Tatsuya Nagano, Sae Murakami, Takashi Omori, Yoshihiro Nishimura, Kazuyuki Kobayashi.

**Validation:** Takehiro Otoshi, Tatsuya Nagano.

**Visualization:** Takehiro Otoshi, Tatsuya Nagano, Sae Murakami, Takashi Omori.

**Writing – original draft:** Takehiro Otoshi, Tatsuya Nagano, Sae Murakami, Takashi Omori.

**Writing – review & editing:** Takehiro Otoshi, Tatsuya Nagano, Sae Murakami, Takashi Omori, Daisuke Hazama, Naoko Katsurada, Masatsugu Yamamoto, Motoko Tachihara, Yoshihiro Nishimura, Kazuyuki Kobayashi.

## Supplementary Material

Supplemental Digital Content
