# Supplementary material for: A randomized crossover pilot study comparing the efficacy of an auto-demand oxygen delivery system with that of a conventional demand oxygen delivery system in patients with chronic respiratory failure
Source: Medicine (Baltimore). 2021 Sep 17;100(37):e27191. doi: 10.1097/MD.0000000000027191 (PMC8448043; doi:10.1097/MD.0000000000027191)

**Supplemental Digital Content**.

Discomfort index questionnaires. Each questionnaire has a score of 1 to 4. The total score (from 5 to 20) is defined as a discomfort index in this study.

251658240
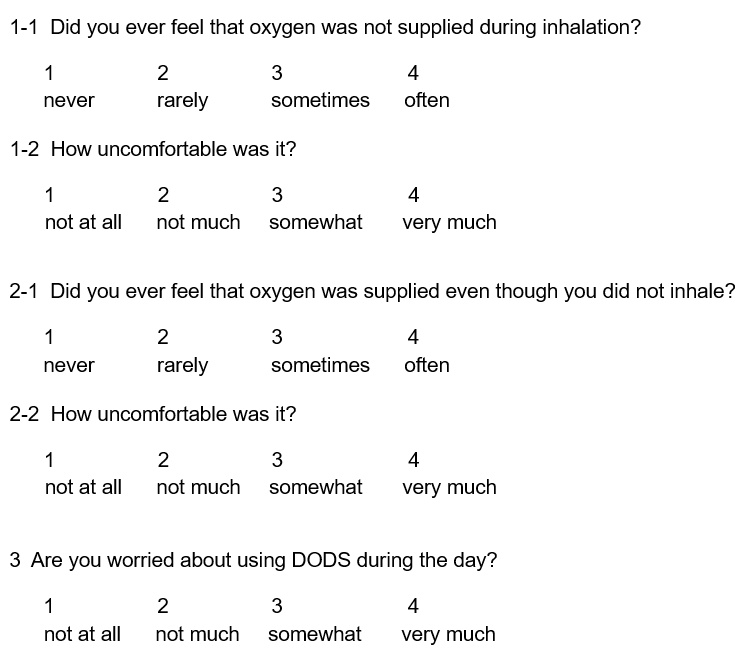

Supplement: Supplemental Digital Content [file medi-100-e27191-s001.doc]
